# A Hybrid DV-Hop Algorithm Using RSSI for Localization in Large-Scale Wireless Sensor Networks

**DOI:** 10.3390/s18051469

**Published:** 2018-05-08

**Authors:** Omar Cheikhrouhou, Ghulam M. Bhatti, Roobaea Alroobaea

**Affiliations:** 1 Department of IT, College of Computers and Information Technology, Taif University, At Taif 26571, Saudi Arabia; 2 Department of Computer Science, College of Computers and Information Technology, Taif University, At Taif 26571, Saudi Arabia; gbhatti@tu.edu.sa (G.M.B.); r.robai@tu.edu.sa (R.A.)

**Keywords:** WSN, localization, DV-Hop, RSSI, IoT, multihop

## Abstract

With the increasing realization of the Internet-of-Things (IoT) and rapid proliferation of wireless sensor networks (WSN), estimating the location of wireless sensor nodes is emerging as an important issue. Traditional ranging based localization algorithms use triangulation for estimating the physical location of only those wireless nodes that are within one-hop distance from the anchor nodes. Multi-hop localization algorithms, on the other hand, aim at localizing the wireless nodes that can physically be residing at multiple hops away from anchor nodes. These latter algorithms have attracted a growing interest from research community due to the smaller number of required anchor nodes. One such algorithm, known as DV-Hop (Distance Vector Hop), has gained popularity due to its simplicity and lower cost. However, DV-Hop suffers from reduced accuracy due to the fact that it exploits only the network topology (i.e., number of hops to anchors) rather than the distances between pairs of nodes. In this paper, we propose an enhanced DV-Hop localization algorithm that also uses the RSSI values associated with links between one-hop neighbors. Moreover, we exploit already localized nodes by promoting them to become additional anchor nodes. Our simulations have shown that the proposed algorithm significantly outperforms the original DV-Hop localization algorithm and two of its recently published variants, namely RSSI Auxiliary Ranging and the Selective 3-Anchor DV-hop algorithm. More precisely, in some scenarios, the proposed algorithm improves the localization accuracy by almost 95%, 90% and 70% as compared to the basic DV-Hop, Selective 3-Anchor, and RSSI DV-Hop algorithms, respectively.

## 1. Introduction

There has been an emerging interest in the field of context-aware computing and location-aware services [1,2,3,4], as a part of the Internet of Things (IoT), where the information on geographical location of the constituent nodes becomes an integral part of the sensor data. As a result, estimating the position of these nodes in the network has become an essential requirement for many applications of wireless sensor networks (WSN). The process of calculating or estimating the position of nodes in wireless sensor networks is known as localization.

Given the scarce resources and unstable operating environments, the localization of wireless sensor nodes is a challenging problem that is currently subject to active research. To localize wireless nodes precisely, efficient localization techniques are required. The traditional approaches to localization, such as the Global Positioning System (GPS)-based methods [5], manual measurement/calibration methods, etc., become infeasible due to a number of reasons including, but not confined to, prohibitively high monetary cost, unsuitability of technology (e.g., GPS-based solutions are not practical for covered/shadowed networks where satellite signals are not directly received), especially for large-scale wireless sensor networks.

In a typical localization scenario, all but a few wireless nodes in the network are initially unaware of their geographic position. Only a handful of wireless nodes, called anchor nodes, posses their location information, either through an automated GPS-based system or manual configuration, after getting deployed. The goal of a localization algorithm is to estimate the position of all non-anchor node (henceforth called sensor nodes) in the network by using the location information of the anchor nodes.

There has been numerous research efforts in the area of localization in WSN and thus many localization algorithms have been proposed in literature during recent years. These can broadly be classified as range-free and range-based localization algorithms. The latter category, as its name implies, is based on the range measurements between the neighboring wireless nodes. These measurements can be realized by using, for example, the time-of-arrival (TOA) method, time-difference of arrival (TDOA), or the received signal strength indicator (RSSI) method. For many researchers, the RSSI-based ranging is a preferable choice due to its relatively low cost. It has thus been widely used for the localization of wireless nodes without a need for the GPS system [6,7,8,9]. However, the RSSI measurements are sensitive to the environmental noise [10] that may lead to a lower localization accuracy.

As compared to range-based localization algorithms, where peer nodes only communicate with their immediate neighboring nodes, the multi-hop localization approach leverages the network topology for estimating the position of sensor nodes that are multiple hops away from the anchor nodes. This approach has the advantage of being able to locate wireless nodes even if these nodes are geographically multiple hops away from the anchors nodes. That may also require a reduced number of anchor nodes in a WSN. However, most multi-hop localization algorithms require strong connectivity between sensor nodes and high-density within the network in order to provide accurate location estimates.

One of the well-known multi-hop localization algorithms is the distance vector-hop (DV-Hop) algorithm [11]. This algorithm works in the following way. It first estimates the average distance per hop between all pairs of anchor nodes. Then, every sensor node estimates its distance from an anchor node by multiplying its hop-count from that anchor node and the average distance per hop. Once a sensor node’s distance from at least three anchor nodes becomes available, one can apply the triangulation method to calculate its estimated location. However, the DV-Hop algorithm results in poor localization accuracy because it uses average distance per hop that might be different from actual distance between the pair of sensor nodes. Moreover, in DV-Hop, if two nodes have the same hop-counts from anchor nodes, both will be assuming the same physical position, which may not be the correct estimate of their locations.

In this paper, we propose an enhanced DV-Hop algorithm, named as Hybrid DV-Hop algorithm, in order to alleviate these problems. The proposed algorithm uses the RSSI measurements between neighbor nodes to estimate distance. Moreover, we also allow the already localized sensor nodes to act as anchors for subsequent localization of remaining sensor nodes. Simulation results show that our localization algorithm outperforms the original DV-Hop algorithm and two of its variants, named as RSSI Auxiliary Ranging [12] and the Selective 3-Anchor DV-hop algorithm [13]. More precisely, in some of our test scenarios, the proposed algorithm improves the localization accuracy by up to 90% as compared to that of the original DV-Hop algorithm.

The remaining paper is organized as follows. We first provide a brief literature review for the reported work related to localization in wireless sensor networks in the next section. The DV-Hop algorithm is described in Section 3. Our proposed algorithm is then presented in Section 4. Our simulations are described in Section 5 that also includes the results from these simulations followed by a discussion on our findings. Finally, the conclusion and direction of our future work are presented in Section 6.

## 2. Related Work

Localization in wireless sensor networks has attracted a large number of researchers during the last years and, as a result, several localization algorithms have been proposed in the literature. These algorithms can broadly be categorized into two classes, i.e., range-based [14] and range-free [15] localization algorithms.

In the former approach, the distance between the target node and its neighboring anchor nodes is estimated by using one of several available techniques such as round-trip time of flight method, time of arrival (TOA) method [16,17,18,19,20], time difference of arrival (TDOA) method [19,21], etc. Once these distances have been determined, the triangulation technique can then be used to estimate the physical position of the target node. It is worth mentioning that every target node must have at least three anchors as its one-hop neighbors for it to be localized in two dimensions (i.e., on a plane). The accuracy of the estimated location, however, depends on how accurately the distances between the target sensor node and its neighboring anchors were calculated. The above mentioned methods, however, require specialized hardware, for example, to provide clock synchronization or ultra wide band (UWB) radios for capturing better signal-arrival time in the wireless sensor nodes in order to achieve a reasonable localization accuracy [22,23]. Another approach, popular with research community, due to its inexpensive and simple nature, is to use the received signal strength indicator (RSSI) for estimating the distance between the transmitter and receiver nodes [6,9,24]. However, this approach seriously suffers from estimation inaccuracies primarily due to the instable nature of the received radio signals. Under the dynamic environmental influences, significant fluctuations in the target metric (e.g., RSSI) take place resulting in large variations in the estimated distances.

The propagation of a wireless signal is mainly influenced by the particular channel being used for transmissions and the specific environment in which it is propagating. Researchers have proposed several methods for improving the accuracy of RSSI-based distance estimates. These RSSI-based localization methods can be classified as those based on fingerprinting and those using signal propagation modeling. The latter aspect requires better propagation model that captures the interference and fading caused by multi-path propagation and shadowing in a specific deployment area [25].

In the former approach, researchers have used the pre-measured distances at specific locations in the deployment area, a process known as fingerprinting [26,27]. The use of fingerprinting normally consist of two phases, i.e., an off-line phase, which is used to carry out RSSI measurements at pre-specified locations in the deployment area, and an on-line phase during which wireless sensor nodes are localized.

The fingerprinting-based localization algorithms, in fact, exploit the RSSI values at the target wireless node as a function of the mobile position as used during the off-line phase. The distances recorded through the fingerprinting phase can then be used for minimizing the effect of fluctuations in the RSSI values, by using, for example, least squared errors method [19] or by matching the actual signature of the RSSI with the corresponding entries stored in a database available at the anchors. The latter localization algorithm consists of DB-assistance, ration base algorithm, and an elementary machine learning algorithm. It was claimed that DB-Assisted Least Error algorithm stabilizes the unstable RSSI values and performs well as compared to traditional RSSI-based algorithms. More specifically, the fingerprints obtained from the database may be used as methods to overcome even some structural obstacles [6].

The fingerprinting can also be used for defining a better transmission loss model that builds a more realistic mathematical model capturing the environmental characteristics of the deployment area. As opposed to manually performed fingerprinting, some researchers have proposed automatic calibration of the wireless propagation model for indoor RSSI-based localization [9]. That approach of estimating the model parameters of path loss in the deployment area might be particularly useful for indoor localization. This approach assigns appropriate weights to the RSSI measurements according to their strength while defining a propagation model to map RSSI measurements into distances specifically in the target deployment area. The resulting model is subsequently used to map RSSI measurements into distances. Specifically, during the localization phase, the target node receives the beacons from anchor nodes, computes the corresponding RSSI, and sends it to a localization server, which, in turn, estimates the mobile node’s position.

Another approach to further improve fingerprinting-based localization is to use feedback from neighboring anchors. Specifically, after the distance between the target and anchor nodes is calculated, the feedback from neighboring anchor nodes is used to improve the accuracy of the calculated distance [7]. Some researchers have proposed a machine-learning-based localization for indoor applications with accesspoint (AP) selection and signal strength reconstruction scheme [8]. It assumes multiple wireless APs being active in the deployed network. It first selects the best anchor (i.e., an AP) based on the best RSSI value and then reconstructs the RSSI values for those anchors for which it has low RSSI values. That enhances algorithm’s robustness against noise. Some researchers have combined RSSI measurements with some auxiliary systems. For example, integrating RSSI measurements with inertial sensors has been claimed to improve the system performance [28].

Although range-based localization can achieve high accuracy, these algorithms require additional hardware (thus increasing the cost) and consume more energy. That bodes not so well for the wireless sensor nodes that are supposed to be low cost and have limited resources. Therefore, several localization solutions have proposed while using range free algorithms. These algorithms aim at estimating the location of a target node without requiring to estimate the pair-wise distances between the wireless nodes. One of the commonly used range free localization algorithm is DV-Hop [11], which exploits the network topology for computing the position of sensor nodes. Specifically, the DV-Hop algorithm first determines the hop-distance (i.e., the number of hops) between pairs of anchor nodes by discovering the shortest paths between them. Since the location coordinates for anchor nodes are pre-specified, these nodes can compute the average distance per hop along these paths by dividing the Cartesian distance between them by the corresponding number of hops. The sensor nodes, which know their hop-distance from anchors, can then use the average distance per hop for computing their estimated distance from every anchor in the network.

Due to the low positioning accuracy of the DV-Hop algorithm, researchers have proposed several improvements in the basic algorithm [12,13,29,30]. For example, an improved twice-refinement DV-Hop localization algorithm that uses the RSSI auxiliary ranging and an error correction mechanism based on target sensor node’s neighborhood centroid is claimed to have improved the localization accuracy [12]. This localization algorithm (referenced in what follows as RSSI DV-Hop algorithm), first estimates every sensor node’s position by using the basic DV-Hop algorithm. It then computes a positioning error model for each sensor node as the difference between the centroid of its neighboring nodes and its estimated position as computed above. This positioning error is then used in a maximum likelihood equation defined based on the distances between the target node and its neighbors using RSSI. In this algorithm, the target node is localized based only on the estimated position of its neighboring nodes without using anchor nodes. In contrast, our algorithm uses both the anchor as well as the neighbor nodes while localizing the target nodes. Moreover, in RSSI DV-Hop algorithm, the error in the estimated position might introduce inaccuracy because the algorithm assumes the centroid of neighboring nodes and the actual position of the node having the same coordinates, which might not necessarily be true.

Moreover, authors in [13] proposed a Selective 3-Anchor DV-hop algorithm. The idea of the Selective 3-Anchor DV-hop algorithm is to use only the 3 best anchors for each node instead of using all connected anchors. The selection of the best anchors is based on the connectivity (number of hops the node is far from anchor) of the node to anchors. In other words, the three nearest anchors (with low number of hops far from the node) will be selected and used for the triangulation.

The authors’ idea is based on the hypothesis that if two sensor nodes have similar connectivities, then they must have similar positions. However, this hypothesis is not always true as nodes might have same connectivity but different positions.

Some researchers aimed at studying the impacts of mobility on DV-Hop-based localization. It was shown that the performance of DV-Hop algorithm is significantly affected by mobility. Consequently, a mobility model was incorporated into the localization algorithm [29].

## 3. DV-Hop Algorithm

The Distance Vector-Hop (DV-Hop) algorithm [11] was proposed as a distributed localization algorithm based on distance vector between nodes. Rather than estimating the distance between neighboring wireless nodes by using ranging methods, this algorithm first calculates the actual distance between every pair of anchor nodes (since the coordinates of these nodes are known a priori). It then finds hop-distance (i.e., the number of hops) between every pair of anchors and calculates the average distance per hop along these paths. These distances are then used for localization of sensor nodes. Specifically, the localization process while using DV-Hop algorithm consists of the following three phases [11,13]:

- **Flooding**: During the first phase, every anchor node, say $A_{i}$, broadcasts a message containing its location coordinates and a hop-count field that is initially set to 0. The hop-count value will be incremented by other nodes every time the message is re-broadcasted.
  When a node *N*, being either anchor or sensor node, receives such a message for the first time, it records the position of $A_{i}$, increments the hop-count value contained in the message, and initializes a local variable $hop_{i}$ by the incremented value of hop-count. Here, $hop_{i}$ represents the minimum number of hops between *N* and $A_{i}$. If another copy of the same message originated from the same anchor $A_{i}$ is later received by *N*, it first retrieves the hop-count value from the just received message, gives it an increment, then compares it with the value of local variable $hop_{i}$. It updates the value of $hop_{i}$ only if the new hop-count value is smaller than the current value of $hop_{i}$ and then broadcasts the message with incremented value of hop-count field. Otherwise, it will simply ignore the message (without even re-broadcasting it). At the completion of this phase, all wireless nodes, anchors and non-anchors, will have their minimum hop distance from every anchor node in the network.
- **Calculating Average Distance/Hop**: During the second phase, each anchor node, say $A_{i}$, after receiving the location coordinates as well as the hop-count values of/from all other anchors, say $A_{j}$, it computes the average distance per hop ($Hopsize_{A_{i}}$) by using the following equation:
  (1) $$Hopsize_{A_{i}}=\frac{\sum_{i\neq j}\sqrt{{\left(x_{i}-x_{j}\right)}^{2}+{\left(y_{i}-y_{j}\right)}^{2}}}{\sum_{i\neq j}hops_{ij}}$$
  where $\left(x_{i},y_{i}\right)$ represents anchor $A_{i}$’s coordinates, and $hops_{ij}$ the number of hops from $A_{i}$ to $A_{j}$. All anchor nodes then broadcast their Hopsize in the network.
- **Estimating Node Position**: After receiving the $Hopsize_{A_{i}}$ values from an anchor node $A_{i}$, a sensor node *N* can compute its estimated distance from the anchor node $A_{i}$ as the following:
  (2) $$distance_{N,Ai}=Hopsize_{A_{i}}\times hop_{i}$$
  where $hop_{i}$ is the number of hops between *N* and $A_{i}$. The sensor node *N* can finally use triangulation [31] to estimate its location after it computes its distance from at least three anchor nodes.

Although the DV-Hop algorithm enjoys the advantages of being simple, computationally less demanding, and low-cost (i.e., without requiring ranging), it suffers from low accuracy, especially in the low density networks. For example, a pair of nodes having the same hop-distance from all anchors (a scenario that is not very uncommon in wireless sensor networks), will have the same estimated position even though the two nodes, in reality, might be located far apart from each other.

In this paper, we propose two improvements to the DV-Hop algorithm aimed at increasing its accuracy. Specifically, we use the RSSI values for estimating the distance between anchor nodes and their immediate neighboring sensor nodes. These estimated distances are then used while localizing these nodes, rather than the average per-hop distances. Moreover, once a sensor node *N* gets localized, we subsequently use it as an anchor node while localizing the remaining sensor nodes. As our results indicate, our enhanced DV-Hop algorithm improves the localization accuracy significantly without sacrificing the simplicity, efficiency, and low-cost nature of the original DV-Hop algorithm.

## 4. Hybrid DV-Hop Algorithm

The RSSI-based localization algorithms, in contrast to their range-based counterparts, are very popular in the research community due to a number of attractive factors. For example, measuring the RSSI and passing it to higher stack layers is a standard feature in the modern wireless sensor nodes. The RSSI-based localization does not require any time-synchronization among nodes, the use of UWB radios for calculating more accurate time of arrival of radio signals, or the use of antenna arrays. It is simple and low-cost method to realize node localization in terms of both software and hardware [4]. On the other hand, the DV-Hop algorithm completely avoids estimating the actual distances between the one-hop neighbor nodes and exploiting these distances for more accurate localization in large scale wireless sensor networks.

As mentioned before, our hybrid algorithm uses two additional steps while localizing wireless nodes using DV-Hop algorithm. In the first step, we use the RSSI values for estimating distances between the anchor nodes and their one-hop neighboring sensor nodes and then use these distances thus estimated rather than using the average hop distance as done in the original DV-Hop algorithm. Since the MAC sub-layer in most of the modern wireless sensor nodes calculates RSSI value for every received packet and passes that value to higher layers, using the RSSI value does not require any special hardware or incur additional costs.

Second, after a sensor node *N* is localized, it is promoted to act as an anchor that is subsequently used for localizing other sensor nodes. The availability of additional (converted) anchor nodes improves the accuracy while localizing the remaining sensor nodes. That is especially helpful in wireless networks with lower anchor node density.

In the following sections, we first present the RSSI-based distance estimation and then explain our proposed method called *Hybrid DV-Hop*.

### 4.1. RSSI Based Distance Estimation

The path loss model of signal propagation is the key to the distance measurement between the transmitter and receiver nodes. It consists of measuring the power present in a radio signal as received at a node and calculating the propagation loss. That loss is then mapped into the distance by using a theoretical or an empirical path loss model. The received signal power of nodes for the log-normal shadow model is defined by [30]:

(3) $$RSS\left(d\right)\left(dBm\right)=P_{tr}-P_{loss}\left(d_{0}\right)-10\tau \log_{10}\frac{d}{d_{0}}+X_{\sigma },$$

where *d* means the distance between the transmitting and receiving nodes, RSS(d) indicates the signal power as received at a node located across a distance of *d* from the transmitting node, $d_{0}$ is the reference distance, $P_{tr}$ denotes the transmitted signal’s power, $P_{loss}\left(d_{0}\right)$ means the signal power loss across the reference distance $d_{0}$, τ is the path loss exponent whose value depends on the medium of propagation, and $X_{\sigma }$ is the noise, which is described as a Gaussian random variable with zero as its mean and σ as the standard deviation.

Our experiments indicate that the estimated distance between neighbor nodes (hop-count = 1) using the RSSI values is more accurate than the average distance per hop as used in DV-Hop algorithm. Therefore, we have used the RSSI values to estimate the distance between the anchor nodes (both the original and the converted anchors) and their immediate neighboring sensor nodes.

### 4.2. Proposed Localization Algorithm

In the basic DV-Hop algorithm, the distance of individual sensor nodes from the anchor nodes are estimated by using average distance per hop. Since there may be significant discrepancy between these estimated and corresponding actual distances, the subsequent step of position estimation of each sensor node by triangulation would introduce large errors in the estimated positions. After doing a set of experiments, we noticed that, for nodes that are one hop away from an anchor, the distance estimated using RSSI is more accurate than the distance estimated using Hopsize. For this reason, in our enhanced algorithm, we propose to localize one-hop sensor node using RSSI-based distance rather than the Hopsize-based distance. Therefore, when a node is directly connected to (i.e., is one-hop neighbor of) at least three different anchors, its location is computed by using triangulation based only on the RSSI-based estimated distances from these anchors. For other nodes, we use anchors and already localized neighbors. Indeed, in our proposed hybrid DV-Hop scheme, once a sensor node is localized, it is used as an anchor node while localizing its neighbor sensor nodes. Using the “converted” anchors too, provides us with additional anchors for use in the triangulation equation that results in an improved localization accuracy.

Moreover, during our experiments, we noticed that the nodes closer to anchors have higher localization accuracy as compared to the nodes farther away from the anchors nodes. In order to exploit that advantage, we proposed that the wireless sensor nodes be localized in a progressive manner such that nodes near anchors are localized first.

The localization process consists of three phases: (1) the Flooding phase; (2) the average distance per hop estimation; (3) the sensor node position estimation. Phase (1) and (2) are done by anchor nodes, however; phase (3) is done by the sensor node to be localized and its different steps are described in Algorithm 1.

The **flooding** phase and the **Calculating Average Distance per Hop** phase are similar to the ones described in Section 3. After the end of these phases, each node knows the list of reachable anchor with their coordinates, their Hopsize, and the number of hop-count away from each reachable anchor.

Then, the localization process of unknown nodes is initiated by the anchors. More precisely, each anchor send a LocStartMsg message to their neighbors in order to start the localization. The LocStartMsg message contains mainly the following field: LocStartMsg={nodeID,Type,nodeCoordinate}, where nodeID represents the identity of the node sending the message, Type specifies either the node is anchor or no and nodeCoordinate the coordinates of the node, which are real coordinates for anchor nodes and estimated coordinates for other sensor nodes. Each node receiving this message, first estimates its position and then forwards the message to their neighbors. By this technique, we ensure that the nodes closest to the anchor nodes are localized first and that the localization is done progressively.

Every node that receives this message memorizes the identity of the neighbor node and its estimated position. Once a node has at least three anchors and/or neighbors, it could compute its location using triangulation.

Note that, every node maintains a LocTable that, initially, contains the list of reachable anchors with their coordinates and Hopsize. This information is obtained from the phases (1) and (2). This table is updated each times the node receives a LocStartMsg message from a neighbor by storing the nodeID and the nodeCoordinate of the neighbor node in this table.

Algorithm 1 summarizes the proposed Hybrid DV-Hop algorithm (phase 3). Step 1 to 9, the sensor nodes estimate the distance to each reachable anchor. As already mentioned, if the anchor is a neighbor to the sensor node (hop-count = 1), then the RSSI technique is used to estimate the distance, otherwise, the distance is estimated using the Hopsize (as in the original DV-Hop algorithm). Then, when a node receives a LocStartMsg (step 10), it first updates its LocTable (step 11). Then the node checks if it has at least three available one-hop anchors. If it is the case, the position is estimated using triangulation and these estimated distances (step 13–14). In the other case, the node will serve with already localized neighbors. For this purpose, it first estimates distance to these neighbors using RSSI (step 18), and then uses triangulation and list of available nodes in LocTable (anchors+neighbors) to estimate its position.

**Algorithm 1** Proposed Algorithm (Hybrid DV-Hop)**Input:** List of reachable Anchors {$A_{i},1<i<n$}, with their coordinates and Hopsize**Output:** Estimated position of unknown node *N* 1: **for** each anchor $A_{i},1<i<n$
**do** 2:  **if**
$A_{i}$ is neighbor to *N* (hopCount==1) **then** 3:   %use RSSI to estimate distance to $A_{i}$ 4:   $distance_{N,Ai}=getDistance\left(RSSI_{N,Ai}\right)$ 5:  **else** 6:   %use $Hopsize_{Ai}$ to estimate distance to $A_{i}$ 7:   $distance_{N,Ai}=Hopsize_{A_{i}}*hop_{i}$ 8:  **end if** 9: **end for** 10: **if** Received LocStartMsg message **then**
 11:  Add nodeID and nodeCoordinate to LocTable. 12:  nbAnch=number of one-hop anchors (hopCount==1) 13:  **if** nbAnch >=3 **then**
 14:   $EstimatedPosition_{N}$=Triangulation ($A_{i},1<i<nbAnch$) 15:  **else**
 16:   nbNeig= number of already localized neighbors 17:   {$Neig_{i},1<i<nbNeig$} = the set of already localized neighbors 18:   Use RSSI to estimate distance to $Neig_{i},1<i<nbNeig$ 19:   $EstimatedPosition_{N}$=Triangulation ($A_{i},1<i<nbAnch$, $Neig_{i},1<i<nbNeig$) 20:  **end if** 21:  Communicate the estimated position of *N* to its neighbors 22: **end if**

The use of neighbors as extra anchors permits to increase the estimation ratio (number of estimated nodes per total number of nodes) as the nodes that are not connected to three anchors (minimum number of input to the triangulation) could not be estimated without using their neighbors as extra anchors. Moreover, the experiments show that the use of extra input to the triangulation function increases the localization accuracy. Finally (step 21), the sensor node sends its estimated position in a LocStartMsg to their neighbors except those from where it already receives a LocStartMsg (as they are already localized).

## 5. Performance Evaluation

In this section, we present our simulation results for the proposed hybrid DV-Hop algorithm. We first offer a brief discussion on the results for three performance parameters and then compare our algorithm’s performance to two existing improvements to the original DV-Hop algorithm. Specifically, we have examined the performance of all four algorithms in terms of three different performance parameters including localization error vs radio transmission range, localization error vs anchor rate (i.e., number of anchors), and localization error vs network density (i.e., number of sensor nodes).

**System Model:** In our first simulation, we have used a wireless sensor network consisting of fixed number of sensor nodes being 100. These nodes were randomly deployed in an area of 100 × 100 m^2^. In order to study the performance of algorithms according to radio transmission range (respectively anchor rate), we select a fixed value of anchor rate (respectively transmission range) and then we vary the value of the transmission range (respectively anchor rate). Also, since both the sensor and anchor nodes in modern WSNs essentially have the same radio, power, and computational (i.e., micro-processor, RAM, ROM, etc.) profiles, we assume all nodes in the network having the similar characteristics. We also assume symmetric links among neighboring nodes, i.e., if node $A_{i}$ can receive a packet transmitted by $A_{j}$, then vice versa is also true. We used Matlab for implementing our simulations.

During these simulations, the number of anchor nodes and the radio transmission range vary in (10%, 20%, 30%, 40%, 50%) and 20 m to 50 m, respectively. Table 1 summarizes the different parameters used in each Figure. For each simulation scenario, we repeat the experiment ten times with new randomly generated nodes locations.

### 5.1. Localization Error vs. Transmission Range

**Figure 1:**
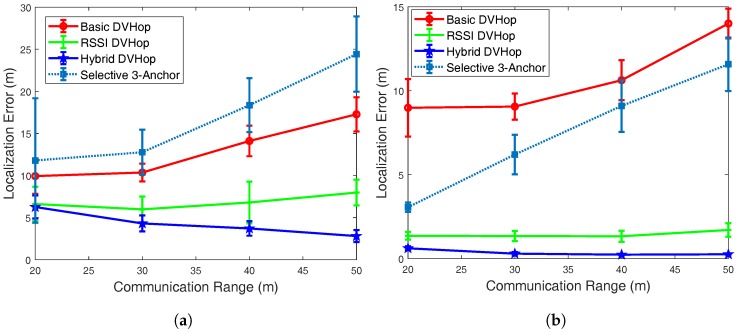
Localization error vs. transmission range. (**a**) Anchors Rate = 10%; (**b**) Anchors Rate = 50%.

The first set of our experiments was aimed at studying the correlation between the radio transmission range and the localization error (in meters) as depicted in Figure 1. Due to the space limit, we targeted two scenarios having different number of anchor nodes. Specifically, the results reported in Figure 1a were achieved by using low level of anchor rate 10% while the system used in Figure 1b had high level of anchor rate 50%. As evident from these plots, for our proposed hybrid DV-Hop algorithm, the localization error reduces as the transmission range increases. This is logical as the increase of the transmission range results in higher number of one-hop anchor nodes and the RSSI factor plays its role in improving the accuracy in the location estimation.

However, for the other DV-Hop schemes the localization error increases with the transmission range. This can be justified by the fact that even sensor nodes that are far from the anchors will reach these anchors and will be estimated, however, they will have relatively large estimation error. In other words, both estimation rate (number of estimated nodes) and estimation error will increase. This problem is avoided in our proposed hybrid DV-Hop scheme as the estimation will be based on neighbor nodes and not far-from anchor nodes.

It is also worth noting that for the systems with lower number of anchors, as shown in (Figure 1a), the Basic DV-Hop scheme outperforms the Selective 3-Anchor scheme as this latter will not have enough anchor to select from them. However, with higher number of anchor (Figure 1b) the Selective 3-Anchor scheme performs better than the basic DV-Hop.

### 5.2. Localization Error vs. Anchor Population

**Figure 2:**
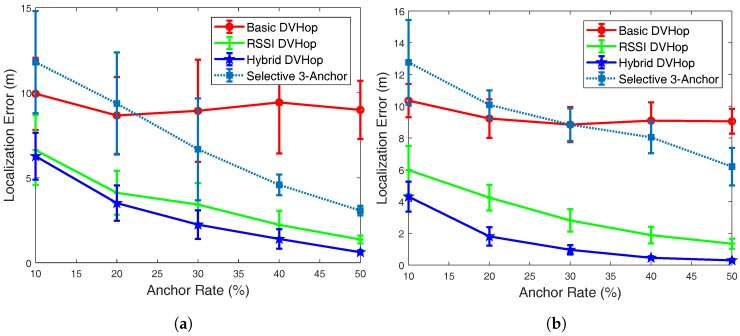
Localization error vs anchor rate. (**a**) Transmission range = 20 m; (**b**) Transmission range = 30 m.

In the second set of simulations, we studied the effect of anchor population on the accuracy of estimation. The results are presented in Figure 2. Again, we targeted two different scenarios; one with transmission range 20 m and the other with 30 m. As expected, the accuracy in the estimated positions improves as the number of anchor nodes in the network increases. Increased number of anchors, in fact, offers more input to the triangulation equation and, thus, results in better precision. It is clear from Figure 2 that the localization error sharply decreases as the number of anchors increases for all the three algorithms (RSSI DV-Hop, Hybrid DV-Hop and Selective 3-Anchor). More precisely, for the case when the transmission range is kept constant at 20 m and the anchor population is varied from 10% to 50%, the localization error decreases from 6.3 m, 6.6 m, and 11.8 m to 0.6 m, 1.3 m, and 3 m for the Hybrid DV-Hop, the RSSI DV-Hop, and the Selective 3-Anchor algorithms, respectively. For the basic DV-Hop scheme, it gives the best result with 20% anchor rate. Then the localization error increases, slightly, with anchor rate. This can be explained that additional anchors will introduce additional error. Moreover, the Hybrid DV-Hop algorithm improves the accuracy of Basic DV-Hop algorithm from almost 50% to 90% when the anchor rate varies from 10% to 50%.

### 5.3. Localization Error vs. Node Density

**Figure 3:**
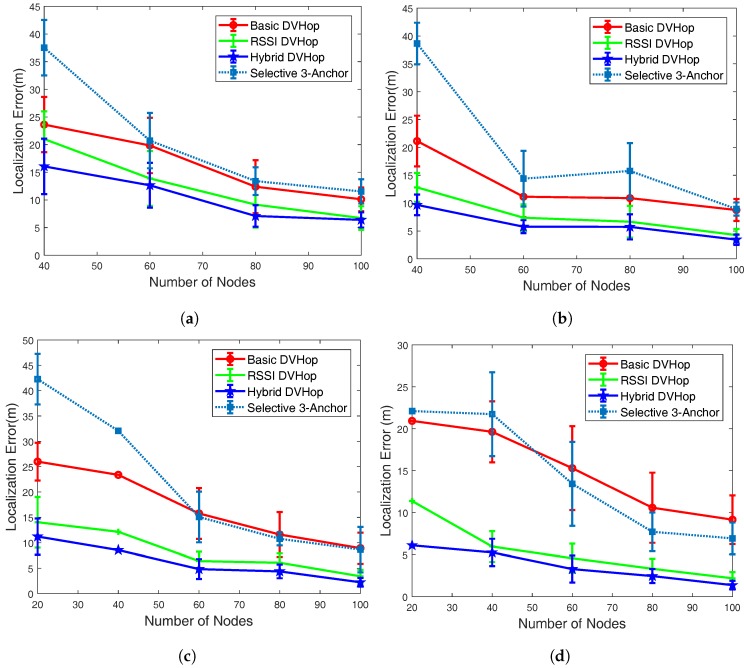
Localization error vs Number of nodes. (**a**) Anchor Rate = 10%; (**b**) Anchor Rate = 20%; (**c**) Anchor Rate = 30%; (**d**) Anchor Rate = 40%.

During these experiments, the number of sensor nodes was varied between 20 and 100 nodes. The transmission range was fixed to 20 m. We targeted four different scenarios while configuring the network with different values of anchor population (i.e., 10%, 20%, 30% and 40% of the total number of sensor nodes). The results are presented in Figure 3. It is interesting to note that as the number of sensor nodes increases, the localization error decreases. That can be attributed to the fact that higher sensor node density results in the increased number of one-hop neighbors and that, in turn, offers increased input to the triangulation function, which produces improved location estimation.

For low value of anchor rate (10% and 20%) and low number of sensor nodes (20 nodes), no node could be estimated and therefore, we could not report result in such case (Figure 3a,b).

For low anchor rate, the basic DV-Hop scheme performs better than the Selective 3-Anchor scheme. This can be justified by the fact that with low number of anchors the scheme has fewer anchors to select between them. For higher value of anchor rate (30% and 40%), the Selective 3-Anchor scheme start performing better than the basic DV-Hop scheme with a higher density of nodes. More precisely, for anchor rate 30% (respectively 40%), when number of node is 60 (respectively 50) the Selective 3-Anchor scheme start performing better than the basic DV-Hop scheme.

### 5.4. Performance Comparison: Hybrid DV-Hop vs. DV-Hop, RSSI DV-Hop and Selective 3-Anchor Algorithms

In order to highlight the performance improvements brought in by our hybrid DV-Hop algorithm, we now compare its performance with that of the basic DV-Hop algorithm and two other variants (improvements) of the DV-Hop algorithm. For this purpose we selected two recently published works, namely RSSI Auxiliary Ranging [12] and the Selective 3-Anchor DV-hop algorithm [13].

As already described in the related work Section, the RSSI DV-Hop scheme is based on RSSI Auxiliary Ranging and the Selective 3-Anchor DV-hop algorithm is based on the selection of the best 3 anchors. As shown in all three previous figures, the proposed hybrid algorithm out-performed both the original DV-Hop as well as the RSSI DV-Hop and the Selective 3-Anchor algorithms in terms of localization accuracy consistently in all three tested scenarios. In particular, the proposed hybrid algorithm offers an improvement to the original DV-Hop one that reaches almost 90% in some scenarios as depicted by Figure 1.

It can also be noticed that, for anchor rate equal to 50% and transmission range equal to 50 m Hybrid DV-Hop improves the localization accuracy of the original DV-Hop, Selective 3-Anchor, and the RSSI DV-Hop algorithms by almost 95%, 90% and 70% respectively (Figure 1b). Moreover, for transmission range equal to 30 m and anchor rate equal to 50%, Hybrid DV-Hop improves the localization accuracy of the original DV-Hop, Selective 3-Anchor, and the RSSI DV-Hop algorithms by almost 94%, 90% and 65% respectively (Figure 2b).

## 6. Conclusions

We have proposed an improved version of DV-Hop localization algorithm, named as Hybrid DV-Hop algorithm, that leverages the RSSI values while localizing the sensor nodes in the immediate neighborhood of the anchor nodes. Since most of the modern wireless sensor nodes offer RSSI value for the received data packets, the proposed algorithm incurs no additional cost in terms of new hardware components or sub-stems. Moreover, the proposed algorithm localizes the sensor nodes in a progressive manner that allows the previously localized sensor nodes to act as anchors while localizing the remaining sensor nodes. We have studied and compared the performance of the proposed algorithm with the basic DV-Hop algorithm and two recently published variants of it, namely RSSI Auxiliary Ranging [12] and the Selective 3-Anchor DV-hop algorithm [13]. The performance evaluation of the four algorithms through simulations indicated that the proposed algorithm significantly outperforms its counterparts. More precisely, the proposed Hybrid DV-Hop algorithm improves the localization accuracy in some scenarios by almost 95%, 90% and 70% as compared to the basic DV-Hop, Selective 3-Anchor, and RSSI DV-Hop algorithms, respectively.

## Figures and Tables

**Table 1:** Simulation parameters.

	Number of Nodes	Anchor Rate	Transmission Range	Environment Dimension
Figure 1	100	10% and 50%	Variable	100 m × 100 m
Figure 2	100	Variable	20 m and 30 m	100 m × 100 m
Figure 3	Variable	10%, 20%, 30% and 40%	20 m	100 m × 100 m

## Abbreviations

The following abbreviations are used in this manuscript:

DV-Hop	Distance Vector Hop
GPS	Global Positioning System
MAC	Media Access Control
RSSI	Received Signal Strength Indicator
WSN	Wireless Sensor Network
